# The Heart, Its Functions

**Published:** 1887-03

**Authors:** 


### THE HEART, ITS FUNCTIONS.

The heart is a bundle of musculine fibers. We know that as long as we live, whether sleeping or waking, that wonderful organ keeps up its regular contractions and expansions. But, when we use our muscles, their contractile force upon the blood vessels helps the blood along its channels, and thus takes a little labor from the propelling heart. It beats faster but with less effort. While helping the heart, muscular exercise helps the lungs also. More exercise means for the lungs more breath ; that is, more air inspired and more carbonic acid gas expired. By deeper breathings the involuntary muscles are strengthened. While the lungs and heart are doing better work under the stimulus of muscular exercise, the heart pumping the blood more certainly to the farthermost tissue of the body and the lungs more rapidly purifying the blood, other organs are benefited, the diaphragm, that muscle separating the lungs and heart from the stomach and liver, is rising and falling, and, with the increased expansion and contraction of the walls of the thorax is moving all the contents of the abdomen to activity. The liver, the great gland of the body, has not only more blood sent to it, but is quickened to action. With each stroke the heart projects something like six ounces of blood into the conduits of the system, and as it does this some 70 times every minute and 4200 times in an hour, this implies that it does the same thing 100,800 times in twenty-four hours, 30,000,000 times in a year, and more than 2,500,000,000 times in a lifetime of 70 years. The mechanical force that is exerted at each stroke amounts to a pressure of thirteen pounds upon the entire charge of blood that has to be pressed onward through the branching network of vessels. According to the lowest estimate that has been made, this gives an exertion of force that would be adequate, in another form of application, to lift 120 tons one foot high every twenty-four hours. Yet the living piece of mechanism that is called upon to do this, and do it without a pause for three score years and ten, without being itself worn out by the effort, is a small bundle of flesh that rarely weighs more than eleven ounces. It is in the nature of the case, also, it must be remembered, that this little vital machine cannot be stopped at any time for repairs. If it gets out of order, it must be set right as it runs. To stop the beating of the heart for more than the briefest interval would be to change life into death. The narrative of what medical science has done to penetrate into the secrets of this delicate force-pump, so jealously guarded from the intrusion of the eye that it cannot even be looked into until its action has ceased, is, nevertheless, a long history of wonders.

